# Incidence, demographics, characteristics and management of acute Achilles tendon rupture: An epidemiological study

**DOI:** 10.1371/journal.pone.0304197

**Published:** 2024-06-21

**Authors:** Samuel Briggs-Price, Jitendra Mangwani, Linzy Houchen-Wolloff, Gayatri Modha, Emma Fitzpatrick, Murtaza Faizi, Jenna Shepherd, Seth O’Neill

**Affiliations:** 1 School of Healthcare, University of Leicester, Leicester, United Kingdom; 2 Orthopaedics, University Hospitals of Leicester, Leicester, United Kingdom; 3 Centre for Exercise and Rehabilitation Sciences, NIHR Biomedical Research Centre, Leicester, United Kingdom; 4 Emergency Care, University Hospitals of Leicester, Leicester, United Kingdom; The University of British Columbia, CANADA

## Abstract

**Background:**

Achilles tendon rupture (ATR) account for 10.7% of all tendon and ligament injuries and causes lasting muscular deficits and have a profound impact on patients’ quality of life. The incidence, characteristics and management of ATR in the United Kingdom (UK) is poorly understood. This investigation aims to understand the incidence of ATR in the UK.

**Methods:**

Prospective data collection of ATR incidence from a United Kingdom Emergency department. Retrospective review of management protocols and immobilisation duration from electronic medical records.

**Results:**

ATR incidence is 8 per 100,000 people per annum. Participants were predominately male (79.2%) and primarily reported a sporting mechanism of injury (65.2%). Mean immobilisation duration was 63.1 days. 97.1% were non-surgically managed post ATR. 46.2% of participants had experienced a previous ATR or Achilles tendinopathy prior to their current ATR.

**Conclusion:**

The incidence of ATR found was 8. cases per 100,000 people per annum. Most ATR were managed non-surgically in this cohort. The majority of ruptures occurred during sporting activity. Almost one quarter (23.3%) of individuals report Achilles pain prior to ATR.

## Background

The Achilles tendon (AT) is the largest and strongest tendon in the body [1]. Through elastic energy storage, the AT improves movement efficiency, transmitting forces of 2.7–3.95 times body weight during walking and 4.15 to 7.71 when running [2]. Achilles tendon ruptures (ATR) can occur when tendon strain exceeds maximum tendon capacity. Common mechanisms for ATR include sudden or violent dorsiflexion of the ankle or a sporting acceleration-deceleration mechanism [3]. ATR are the most common tendon ruptures accounting for 10.7% of all tendon and ligament injuries [4]. Incidence rates range is 2.5–47 per 100,000 person-years in north America and Europe [5–10]. ATR incidence is rising, with the most significant increase between the ages of 40–59 [6, 7]. Significant variation in incidence occurs due to the population sampled (male/female), sample age, geographic range (local/regional/national), sampling setting (emergency department/medical database review) or time of sampling (season). UK ATR incidence increased from 6–13 per 100,00 person years from 1995 to 2019 [11, 12]. However, this data represents ATR incidence in Scotland and a single NHS trust in England. Further incidence data is required to improve understanding of ATR incidence in England.

Risk factors for ATR can be categorised as intrinsic and extrinsic. Intrinsic factors include AT properties, age, sex, genetics and systemic comorbidities. Extrinsic factors include sporting activity, exposure to AT loading and medications [13, 14]. Previous studies have proposed that the rising incidence of ATR is associated with the increasing use of medications associated with ATR in an active, aging population [15]. However, the quality of evidence remains low and further research is required to understand the demographics of the ATR population in the UK.

Surgical and non-surgical management approaches following ATR have been compared extensively. In the UK, non-surgical approaches represent standard practice due to comparable rates of re-rupture with accelerated functional rehabilitation protocols and lower associated complications [16–20]. Recent randomised controlled trials have reported an increased re-rupture risk following non-surgical management [21]. However, in this trial, non-surgical management did not represent the typical accelerated functional rehabilitation non-surgical management protocols that have been developed in the UK, such as the Leicester Achilles Management Protocol (LAMP) and Swansea Morriston Achilles Rupture Treatment (SMART) protocol [22, 23]. AT re-ruptures are reported between 0.9–2% when adopting these non-surgical protocols. However, due to significant loss to follow up, the studies investigating these protocols may under-represent non-surgical re-rupture rates and should be interpreted with caution.

This investigation aims to 1) identify the incidence of ATR in the UK 2) identify the characteristics of the ATR population 3) Identify rates of surgical and non-surgical management including duration of boot immobilisation.

## Methods

### Trial design

Retrospective analysis of prospectively collected data of individuals presenting to the emergency department (ED) diagnosed with ATR. Data was collected at a National Health Service (NHS) Trust from March 2015 to June 2021.

### Participants

**Fig 1:**
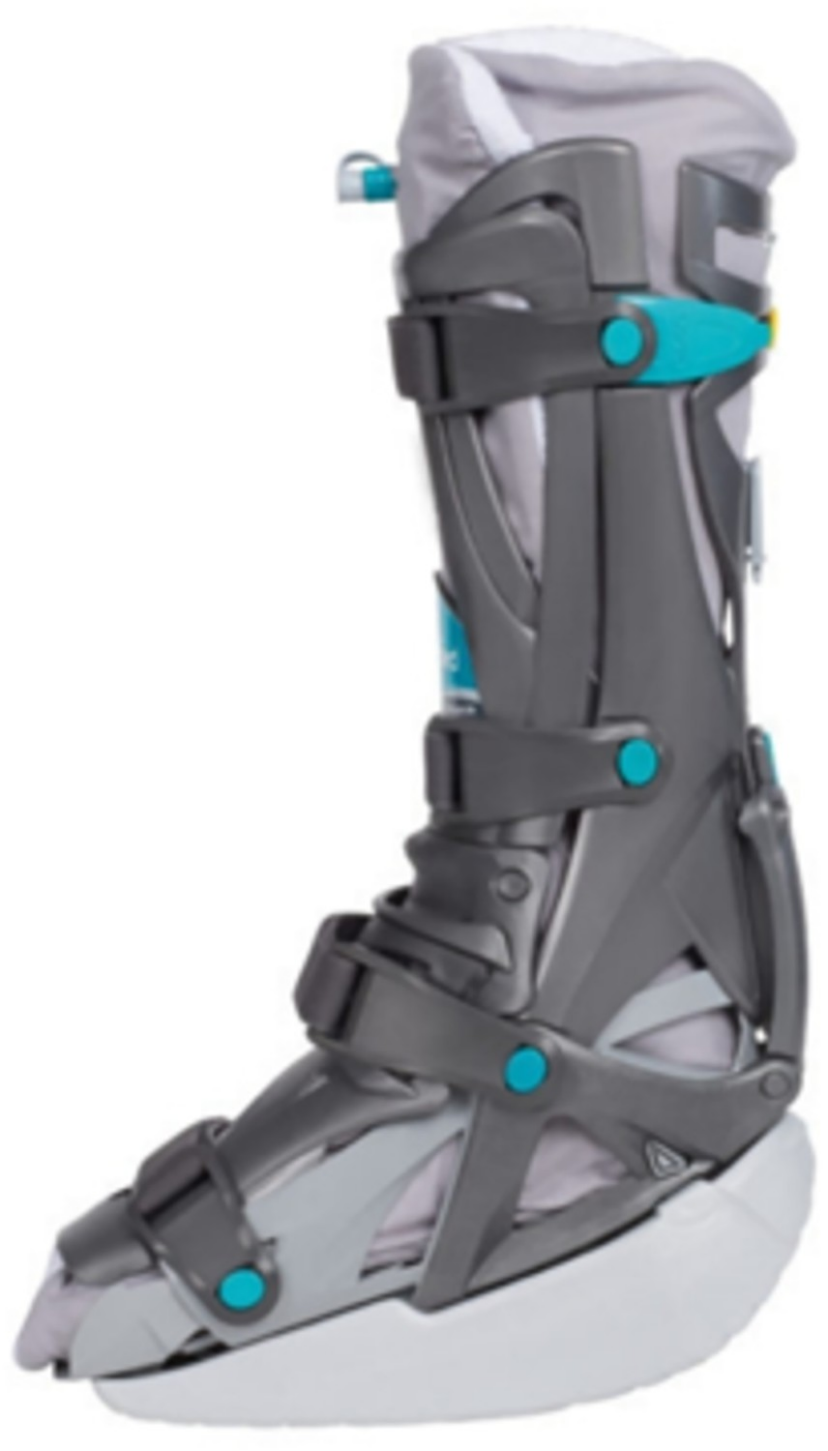
Vacoped controlled mobilisation boot.

All individuals with clinically confirmed ATR documented in ED medical notes were included in the analysis. Comorbidity and medication data was extracted from ED medical notes. In addition to routine comorbidity data collection, participants were asked if they had experienced previous Achilles injury or pain. Medical records were reviewed retrospectively to determine management protocols (surgical/non-surgical) and controlled mobilisation (VACOped™ boot) (Fig 1) duration. The NHS trust studied routinely uses the Leicester Achilles Management Protocol (LAMP) [22] consisting of 8 weeks-controlled mobilisation.

### Data analysis

Data was analysed using SPSS (V28.0, IBM, New York, USA). Data distribution was assessed for normality and reported as means/ median with standard deviation (SD)/ interquartile range (IQR).

Annual incidence data was calculated using complete years. Population data was taken from the County Council demography report [24]. Comorbidity/ medication data used for comparison was taken from NHS or musculoskeletal health reports [25–27].

The relationship between mechanism of injury, demographics, comorbidities and medications are explored. The management approach (surgical/non-surgical) was analysed in relation to participant demographics, comorbidities and duration to present to ED. Groups were compared using an independent t-test or non-parametric equivalent.

## Results

There were 361 participants diagnosed with ATR in ED and included in analysis. Participant’s median (IQR) age was 45 (19) years and were predominantly male (79.2%). Comorbidity and medication data was available for 328 and 316 participants. Comorbidities and medications documented on ED assessment and the comparison to local prevalence are displayed in Tables 1 and 2. Concurrent with routine data collection, pre-rupture Achilles pain/ injury data was collected from 117/361 participants. Achilles pain/ injury prior to ATR was reported by 46.2% (n = 54/117) of participants (Table 3). In participants with Achilles pain/ injury, 61.1% (n = 33/54) reported their complaint bilaterally or on the side of current ATR. There was no significant difference in the number of comorbidities between participants who reported previous AT pain or rupture and participants with no prior symptoms.

**Table 1:** Number and classification of comorbidities.

	ATR Participants (n = 328)	General Population
Number of Comorbidities Mean (SD)	1.2 (1.5)	
Diabetes n (%)	22 (6.9)	6.5%
Hypertension n (%)	40 (12.6)	13.8%
Cardiovascular Disease n (%)	33 (10.3)	9.1%
Rheumatological condition n (%)	3 (1.0)	0.7%
Respiratory n (%)	31 (9.8)	7.8%
Other Musculoskeletal n (%)	70 (22.1)	32%

**Table 2:** Number and classification of medications.

	ATR Participants (n = 316)		General Population
Number of Medications Mean (SD)	1.1 (1.8)		
Statin n (%)	37 (11.7)		14%
Antihypertensive n (%)	46 (14.6)		15%
Analgesia n (%)	14 (4.4)		11%
Proton Pump Inhibitors n (%)	18 (5.7)		11%
Steroids n (%)	29 (9.2)	Inhaled:17 (58.6)	
		Injected: 5 (17.2)	
		Oral:7 (24.1)	
Antibiotics (fluoroquinolones) (%)	2 (0.6)		
Anticoagulants (%)	5 (1.6)		5%
Anti-inflammatories (%)	13 (4.1)		11%

**Table 3:** Achilles pain/injury pre-rupture.

Achilles Pain/ Injury	n (%)
Contralateral Achilles Rupture (n = 117)	19 (16.2)
Ipsilateral Achilles Rupture (n = 117)	7 (6.0)
Contralateral Achilles Tendinopathy (n = 112)	2 (1.8)
Ipsilateral Achilles Tendinopathy (n = 112)	16 (14.3)
Bilateral Achilles Tendinopathy (n = 112)	10 (8.9)
Total participants reporting Achilles Pain/Injury Pre-Rupture (n = 117)	54 (46.2)

The primary mechanism of injury was sporting activities (65.2%). Participants with a sporting mechanism of injury had a mean (SD) age of 41.0 (12.2) years in comparison to 55.4 (13.4) in participants with a non-sporting mechanism of injury (p = 0.55). Participants with a sporting mechanism of injury had statistically significantly fewer comorbidities (0.9 (1.3) vs 1.7 (1.8), p = <0.001) and medications (0.7 vs 1.7, p = <0.001) than those with a non-sporting mechanism of injury.

The median (IQR) time to present to ED following injury was 0 days (1). Nearly all (97.1%) of the participants were non-surgically managed post ATR. There were no significant differences in age or number of comorbidities between surgical and non-surgical groups. The surgical group had a greater mean (days) duration between initial injury and presenting to ED (2.6 (4.9) vs 1.9 (4.8), p = 0.36). The mean (SD) duration wearing the immobilisation boot was 63.1(10.8) days.

**Fig 2:**
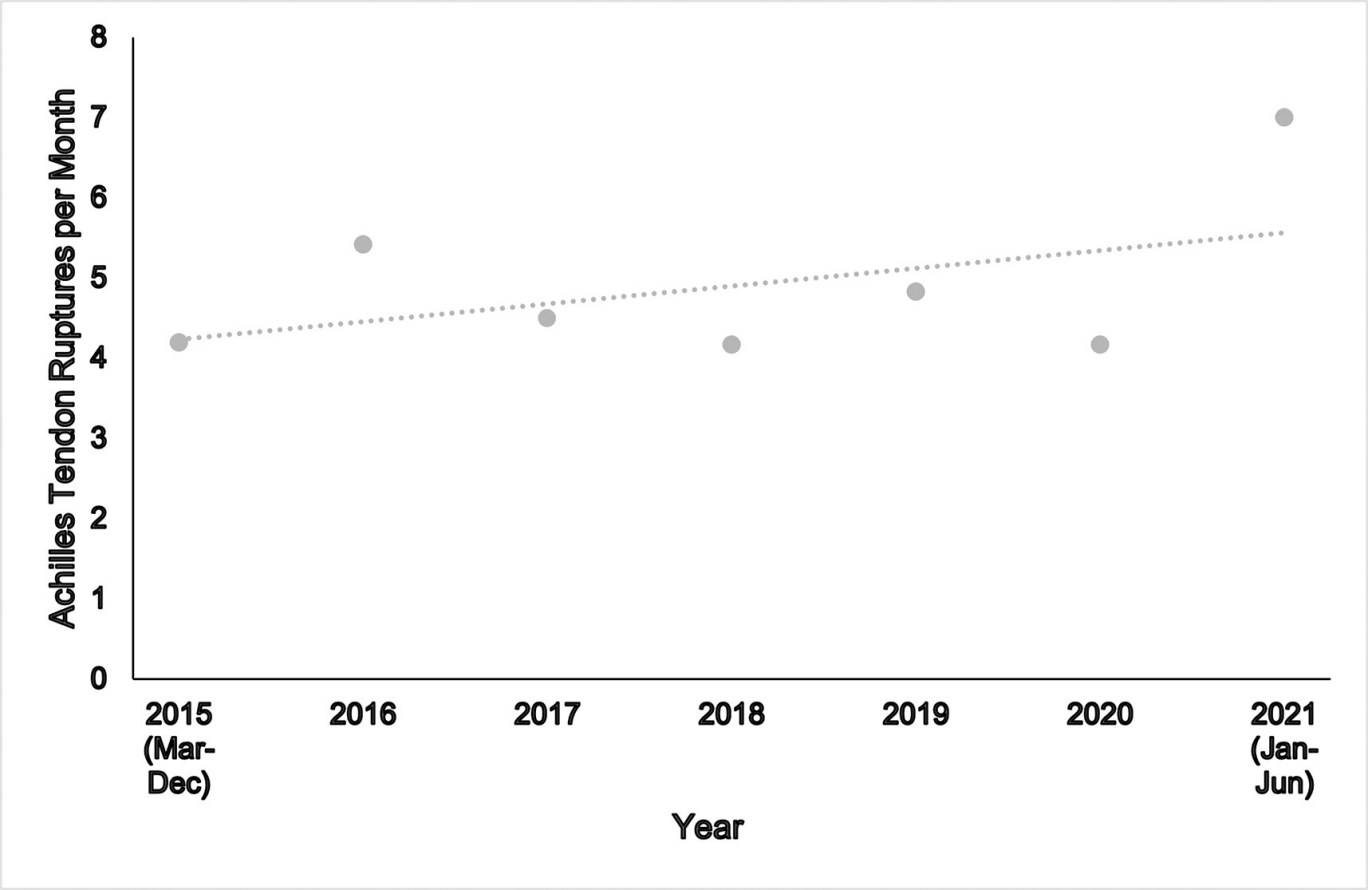
Achilles tendon rupture incidence per month.

The ATR per month incidence rate for each year (March 2016-June 2021) is provided in Fig 2 for all participants.

Annual ATR incidence was calculated for complete years (2016–2020, n = 277). The mean (SD) annual incidence was 56 (6) ATR per year. An incidence rate of 8 per 100,000 people per annum.

The reference line shows the line of best fit over the last 6 years of ATR data.

## Discussion

The incidence of ATR presenting to ED was 8 per 100,000 people per annum. Consistent with previous UK ATR data, an increasing incidence of ATR was identified [11]. International ATR incidence rates are varied, these findings report a higher incidence than the United States, 3.2 per 100,000 person years [7] but lower than other nations (29.5–32.3 per 100,000 person years) [5, 6]. Incidence reports in the United States represent data from an electronic database review [7]. As all medical centres were not included, this review is unlikely to capture all ATR and is expected to under-represent the true incidence rate in the United States.

The ATR population is predominantly male, and injuries most commonly occur during sporting activities. This is consistent with current incidence studies [5–7]. However, males represented a greater proportion of participants and ATR were more frequently associated with non-sporting mechanisms than previously observed [7, 28].

Medications and comorbidities may contribute to ATR aetiology [13, 14]. The overall distribution of comorbidities and medications were similar to the general population. Fluroquinolones were only reported in two cases despite the known association with ATR [29]. The medication dose and interaction between concomitant medications was not investigated and may have a significant impact on ATR risk. The lack of comorbidities and medications found may be due to ATR primarily occurring in the sporting population who present with less comorbid health conditions. In this study, the non-sporting population are older and have greater number of comorbidities and medications. Identifying an older subgroup who present with greater number of comorbidities may provide an insight into tailoring future immobilisation and rehabilitation protocols.

Previous ATR or pain was reported in nearly half of participants. This is the first UK data on Achilles symptoms prior to rupture. As data collection was additional to usual care the sample was limited and findings should be interpreted with caution. In comparison, previous studies found one third of participants experience Achilles symptoms prior to ATR [30]. Achilles tendinopathy is a risk factor for ATR with 4% of individuals with Achilles tendinopathy experiencing ATR [14, 30]. It is unknown if current interventions for AT pathologies reduce the risk of ATR as the ability to alter tendon structure is debated [31].

The UK management of ATR is predominantly non-surgical. This contrasts with surgical management rates seen internationally [5, 6]. The immobilisation duration is consistent with published protocols in this region of the UK [22]. There is a disparity with other regions in the UK which adopt longer immobilisation periods [23].

### Study limitations

This study was limited to a single ED site, it is expected that primary care sites across the region that were not included in this analysis will manage acute ATR. Therefore, the incidence reported is conservative and ATR presenting to secondary care is anticipated to be higher. Future research needs to include larger numbers of primary care sites across the UK to determine the true incidence rate and management of ATR. Understanding current ATR incidence and management is essential for the development of future care in this population.

## Conclusion

The incidence rate of ATR in England is higher than previously reported elsewhere in the UK. There is a continuing trend towards increasing ATR incidence each year. Non-sporting mechanisms of injury are more common than previously reported and occur in an older population with greater number of comorbidities and medications.

## List of abbreviations

AT: Achilles Tendon
ATR: Achilles Tendon Rupture
UK: United Kingdom
LAMP: Leicester Achilles Management Protocol
SMART: Swansea Morriston Achilles Rupture Treatment
ED: Emergency Department
NHS: National Health Service
SD-: Standard Deviation
IQR: Interquartile Range

## References

[1] O’Brien M. The Anatomy of the Achilles Tendon 2005;10:225–38. 10.1016/j.fcl.2005.01.011. 15922915

[2] Demangeot Y, Whiteley R, Gremeaux V, Degache F. The load borne by the Achilles tendon during exercise: A systematic review of normative values 2022. 10.1111/sms.14242. 36278501

[3] Tarantino D, Palermi S, Sirico F, Corrado B. Achilles Tendon Rupture: Mechanisms of Injury, Principles of Rehabilitation and Return to Play. Journal of functional morphology and kinesiology 2020 Dec 17,;5(4):95–0. doi: 10.3390/jfmk5040095 33467310 PMC7804867

[4] Clayton RAE, Court-Brown CM. The epidemiology of musculoskeletal tendinous and ligamentous injuries 2008;39:1338–44. doi: 10.1016/j.injury.2008.06.021 19036362

[5] Leino O, Keskinen H, Laaksonen I, Mäkelä K, Löyttyniemi E, Ekman E. Incidence and Treatment Trends of Achilles Tendon Ruptures in Finland: A Nationwide Study 2022;10:23259671221131536. 10.1177/23259671221131536. PMC9647260 36389616

[6] Sheth U, Wasserstein D, Jenkinson R, Moineddin R, Kreder H, Jaglal SB. The epidemiology and trends in management of acute Achilles tendon ruptures in Ontario, Canada: a population-based study of 27 607 patients 2017:78–86. 10.1302/0301-620X.99B1.BJJ-2016-0434.R1. 28053261

[7] Lemme NJ, Li NY, DeFroda SF, Kleiner J, Owens BD. Epidemiology of Achilles Tendon Ruptures in the United States: Athletic and Nonathletic Injuries From 2012 to 2016 2018;6:2325967118808238. 10.1177/2325967118808238. PMC6259075 30505872

[8] Andrej Čretnik, Roman Košir. Incidence of Achilles tendon rupture: 25-year regional analysis with a focus on bilateral ruptures. Journal of international medical research 2023 Nov 1,;51(11):3000605231205179. doi: 10.1177/03000605231205179 37976267 PMC10657533

[9] Ganestam A, Kallemose T, Troelsen A, Barfod KW. Increasing incidence of acute Achilles tendon rupture and a noticeable decline in surgical treatment from 1994 to 2013. A nationwide registry study of 33,160 patients. Knee Surg Sports Traumatol Arthrosc 2016 Dec 1,;24(12):3730–3737. 10.1007/s00167-015-3544-5 25697284

[10] Huttunen T.T., Kannus P., Rolf C., Felländer-Tsai L. & Mattila V.M. 2014, "Acute achilles tendon ruptures: incidence of injury and surgery in Sweden between 2001 and 2012", The American journal of sports medicine, vol. 42, no. 10, pp. 2419–2423. doi: 10.1177/0363546514540599 25056989

[11] Maffulli N, Waterston S, Squair J, Reaper J, Douglas S. Changing Incidence of Achilles Tendon Rupture in Scotland: A 15-Year Study 1999;9:157–60. 10.1097/00042752-199907000-00007. 10512344

[12] Carmont MR, Morgan F, Fakoya K, Heaver C, Brorsson A, Nilsson-Helander K. The influence of the COVID pandemic on the epidemiology of Achilles tendon ruptures in east Shropshire, United Kingdom. Journal of ISAKOS 2023 Apr 1,;8(2):94–100. doi: 10.1016/j.jisako.2022.10.002 36375752

[13] Claessen F, de Vos R-J, Reijman M, Meuffels D. Predictors of Primary Achilles Tendon Ruptures 2014;44:1241–59. 10.1007/s40279-014-0200-z. 24929701

[14] Xergia SA, Tsarbou C, Liveris NI, Hadjithoma Μ, Tzanetakou IP. Risk factors for Achilles tendon rupture: an updated systematic review 2022:1–11. 10.1080/00913847.2022.2085505. 35670156

[15] Nyyssönen T, Lantto I, Lüthje P, Selander T, Kröger H. Drug treatments associated with Achilles tendon rupture. A case‐control study involving 1118 Achilles tendon ruptures. Scandinavian journal of medicine & science in sports 2018 Dec;28(12):2625–2629. doi: 10.1111/sms.13281 30120842

[16] Reda Y, Farouk A, Abdelmonem I, El Shazly OA. Surgical versus non-surgical treatment for acute Achilles’ tendon rupture. A systematic review of literature and meta-analysis 2020;26:280–8. 10.1016/j.fas.2019.03.010. 31027878

[17] Zhou K, Song L, Zhang P, Wang C, Wang W. Surgical Versus Non-Surgical Methods for Acute Achilles Tendon Rupture: A Meta-Analysis of Randomized Controlled Trials 2018;57:1191–9. 10.1053/j.jfas.2018.05.007. 30368430

[18] Zhang H, Tang H, He Q, Wei Q, Tong D, Wang C, et al. Surgical Versus Conservative Intervention for Acute Achilles Tendon Rupture: A PRISMA-Compliant Systematic Review of Overlapping Meta-Analyses 2015;94:e1951. 10.1097/MD.0000000000001951. PMC4912260 26559266

[19] Deng S, Sun Z, Zhang C, Chen G, Li J. Surgical Treatment Versus Conservative Management for Acute Achilles Tendon Rupture: A Systematic Review and Meta-Analysis of Randomized Controlled Trials 2017;56:1236–43. 10.1053/j.jfas.2017.05.036. 29079238

[20] She G., Teng Q., Li J., Zheng X., Chen L. & Hou H. 2021, "Comparing Surgical and Conservative Treatment on Achilles Tendon Rupture: A Comprehensive Meta-Analysis of RCTs", Frontiers in surgery, vol. 8, pp. 607743. doi: 10.3389/fsurg.2021.607743 33681281 PMC7931800

[21] Myhrvold SB, Brouwer EF, Andresen TKM, Rydevik K, Amundsen M, Grün W, et al. Nonoperative or Surgical Treatment of Acute Achilles’ Tendon Rupture. The New England journal of medicine 2022 Apr 14,;386(15):1409–1420. doi: 10.1056/NEJMoa2108447 35417636

[22] Aujla RS, Patel S, Jones A, Bhatia M. Non-operative functional treatment for acute Achilles tendon ruptures: The Leicester Achilles Management Protocol (LAMP) 2019;50:995–9. 10.1016/j.injury.2019.03.007. 30898390

[23] Hutchison AM, Topliss C, Beard D, Evans RM, Williams P. The treatment of a rupture of the Achilles tendon using a dedicated management programme 2015:510–5. 10.1302/0301-620X.97B4.35314. 25820890

[24] Leicestershire Joint Strategic Needs Assessment 2018–2021 Demography Report. Leicestershire County Council; 2021.

[25] Health Survey for England 2016. Health, social care and lifestyles Summary of key findings 2018. https://www.publicinformationonline.com/key-non-parliamentary-papers/central-government/2018/9781787340992/158357.

[26] Health Survey for England 2016. Prescribed Medicines Health Survey 2017. http://healthsurvey.hscic.gov.uk/media/63790/HSE2016-pres-med.pdf.

[27] Arthritis Versus, The State of Musculoskeletal health 2021 Arthritis and other musculoskeletal conditions in numbers 2021.

[28] Lantto I, Heikkinen J, Flinkkilä T, Ohtonen P, Leppilahti J. Epidemiology of Achilles tendon ruptures: Increasing incidence over a 33-year period 2015;25:e133–8. 10.1111/sms.12253. 24862178

[29] Morales D.R., Slattery J., Pacurariu A., Pinheiro L., McGettigan P. & Kurz X. 2019, "Relative and Absolute Risk of Tendon Rupture with Fluoroquinolone and Concomitant Fluoroquinolone/Corticosteroid Therapy: Population-Based Nested Case–Control Study", Clinical drug investigation, vol. 39, no. 2, pp. 205–213. doi: 10.1007/s40261-018-0729-y 30465300 PMC6394638

[30] Yasui Y, Tonogai I, Rosenbaum AJ, Shimozono Y, Kawano H, Kennedy JG. The Risk of Achilles Tendon Rupture in the Patients with Achilles Tendinopathy: Healthcare Database Analysis in the United States 2017;2017:7021862–4. 10.1155/2017/7021862. PMC5429922 28540301

[31] Docking SI, Cook J. How do tendons adapt? Going beyond tissue responses to understand positive adaptation and pathology development: A narrative review 2019;19:300–10. PMC6737558 31475937

