# Supplementary material for: Incidence, demographics, characteristics and management of acute Achilles tendon rupture: An epidemiological study
Source: PLoS One. 2024 Jun 21;19(6):e0304197. doi: 10.1371/journal.pone.0304197 (PMC11192343; doi:10.1371/journal.pone.0304197)
Supplement: Attachment — Submitted filename: Response to Reviewers.docx [file pone.0304197.s001.docx]

Thank you for taking the time to review our manuscript. Please see below for a point-by-point response to feedback

| Reviewer 1 | | |
| --- | --- | --- |
| 1 | The Title may be misleading: I would specifically report that this is an epidemiological study. | Added as suggested |
| 2 | 2) Introduction is short but fine. You may add something more on how an ATR can occur, focusing on what kind of specific movements (eg. accelerating after landing with violent ankle dorsiflexion) and comorbidities/drugs (eg. diabetes, fluoroquinolones, etc). This could be helpful for a better uderstantind of your results. | Added as suggested (Line 6-7, Line 24-26). |
| 3 | Methods: it should be reported "who made what". | It is unclear what the reviewer means and therefore no modifications have been made |
| 4 | Methods: a Figure of the VACOped boot and a biref description could be useful. | Added as suggested (Line 56-59). Additional figure added displaying the Vacoped boot |
| 5 | Results: Figure 1 is missing. | Figure 1 was submitted as an additional file titled ‘figure 1’ on the submission portal. |
| 6 | Discussion: I suggest to give short interpretation of your results, linking them to some specific observed conditions or drugs (eg. diabetes, higher statins consumption, sterioids, antibiotics, etc). | Added as suggested (Line 151-167). |
| 7 | Please consider to cite the following articles:  - Tarantino D, Palermi S, Sirico F, Corrado B. Achilles Tendon Rupture: Mechanisms of Injury, Principles of Rehabilitation and Return to Play. J Funct Morphol Kinesiol. 2020 Dec 17;5(4):95. doi: 10.3390/jfmk5040095. PMID: 33467310; PMCID: PMC7804867.  - Tarantino, D., Palermi, S., Sirico, F., Balato, G., D‘Addona, A., & Corrado, B. (2020). Achilles tendon pathologies: How to choose the best treatment. Journal of Human Sport and Exercise, 15(4proc), S1300-S1321. doi:https://doi.org/10.14198/jhse.2020.15.Proc4.29  - Tarantino D, Mottola R, Resta G, Gnasso R, Palermi S, Corrado B, Sirico F, Ruosi C, Aicale R. Achilles Tendinopathy Pathogenesis and Management: A Narrative Review. Int J Environ Res Public Health. 2023 Aug 30;20(17):6681. doi: 10.3390/ijerph20176681. PMID: 37681821; PMCID: PMC10487940. | Thank you for these suggestions. The first article has been referenced (Line 6-7). A proportion of the primary research referenced in the second review has been discussed. The third article has not been referenced due to the addition of the previous narrative review and as it primarily discusses Achilles tendinopathy. |
| Reviewer 2 | | |
| 1 | Well written. But why do you not state “symptomatic” instead of “asymptomatic” in the last sentence in the conclusion? Most ATR occur in individuals with no prior pain before rupture… | The wording of statement in the abstract conclusion has been edited for clarity. |
| 2 | Line 7-8: Regarding incidences in north America and Europe. The study by Lemme et al (ref. 6) used data from the National Electronic Injury Surveillance System (NEISS) database which collects data from approximately 100 representative US hospitals with at least 6 beds and with a 24-hours emergency department services. Achilles tendon ruptures treated outside these kinds of hospitals (non-operatively or surgically) will not be registered in the database. Therefore, the data from the Lemme study is most probably not representative for the true incidence of acute Achilles tendon rupture in the US. The incidence is most probably higher. The observed increase in incidence, however, is probably realistic. Suggest to either comment on this or remove the reference and the incidence numbers referred to in the text.  Or consider using the article from Cretnik instead of Lemme and narrow the incidence interval according to the incidence in the remaining publications (Čretnik A, Košir R. Incidence of Achilles tendon rupture: 25-year regional analysis with a focus on bilateral ruptures. J Int Med Res. 2023 Nov;51(11):3000605231205179. doi: 10.1177/03000605231205179. PMID: 37976267; PMCID: PMC10657533.) | Thank you for this guidance. A comment on the variation between incidence rate depending on population, age, geographic region, sampling setting and time of sampling has been added (Line 11-14). An additional comment has been made in the discussion regarding the sample used in the study conducted by Lemme (See comment 8)  The study by Cretnik has been included as an additional reference for the European cohort (Line 10). The study conducted by Lemme has been included to increase representation of incidence in north America. |
| 3 | The authors might also consider using this reference: Ganestam A, Kallemose T, Troelsen A, Barfod KW. Increasing incidence of acute Achilles tendon rupture and a noticeable decline in surgical treatment from 1994 to 2013. A nationwide registry study of 33,160 patients. Knee Surg Sports Traumatol Arthrosc. 2016 Dec;24(12):3730-3737. doi: 10.1007/s00167-015-3544-5. Epub 2015 Feb 20. PMID: 25697284 | Added as suggested (Line 10) |
| 4 | Line 18-21: The authors claim there is similar risk of re-rupture comparing surgical and non-surgical treatment. This is most probably an assumption based on RCTs wich are under-powered (ref. 10-13). The largest RCT performed on treatment results following ATR clearly finds a significant higher incidence of re-rupture after non-operative treatment (Myhrvold SB, Brouwer EF, Andresen TKM, Rydevik K, Amundsen M, Grün W, Butt F, Valberg M, Ulstein S, Hoelsbrekken SE. Nonoperative or Surgical Treatment of Acute Achilles' Tendon Rupture. N Engl J Med. 2022 Apr 14;386(15):1409-1420. doi: 10.1056/NEJMoa2108447. PMID: 35417636.) | Thank you for your comment regarding the sample limitations of the given references. The study referenced (Myhrvold 2022) has been included to report the increased re-rupture risk of non-surgical management (Line 31-33). However, this study consists of a dated method of non-surgical management and does not clearly describe the post-boot rehabilitation programme. Therefore, in isolation, it is not considered a decisive study comparing re-rupture rates. |
| 5 | I would also suggest the authors to read the editorial of Barfod and Hölmich stating that surgery protects from re-rupture (Barfod KW, Hölmich P. Acute Achilles' Tendon Rupture - Surgery or No Surgery. N Engl J Med. 2022 Apr 14;386(15):1465-1466. doi: 10.1056/NEJMe2202696. PMID: 35417642.) | Thank you for this suggestion. This has been considered when developing the manuscript. |
| 6 | Tables 1, 2 and 3:  Please remove the “%” from the parentheses in the second column. | Edited as suggested |
| 7 | Line 129: Please replace “prevalence of ATR” with “incidence of ATR”. | Edited as suggested (Line 140) |
| 8 | Line 130: Again (as in line 7-8): The low incidence data from US is not trustworthy for the true incidence of ATR. Suggest to either comment on this or rephrase. | The limitations highlighted in comment 2 have been included in the discussion (Line 143-146) |
| 9 | Secondly ref. 15 (Hutchison). This is a study which should be interpreted with a high degree of caution. I suggest the authors to read the answer to the Editor in tbe NEJM from 2022 (Myhrvold SB, Ulstein S, Hoelsbrekken SE. Nonoperative or Surgical Treatment of Acute Achilles' Tendon Rupture. Reply. N Engl J Med. 2022 Jul 7;387(1):91. doi: 10.1056/NEJMc2206333. PMID: 35793214.) Please take this into consideration and adapt this knowledge of potential loss to follow-up in that study when referring to this throughout the manuscript. Or consider removing the reference. | The loss to follow up in these studies and the impact on re-rupture rates reported have been acknowledged and added when referencing these studies (Line 37-39) |
