# Supplementary material for: Incidence, demographics, characteristics and management of acute Achilles tendon rupture: An epidemiological study
Source: PLoS One. 2024 Jun 21;19(6):e0304197. doi: 10.1371/journal.pone.0304197 (PMC11192343; doi:10.1371/journal.pone.0304197)
Supplement: Attachment — Submitted filename: Response to Reviewers.docx [file pone.0304197.s002.docx]

| Reviewer 2 | | |
| --- | --- | --- |
| (1) | Minor concern: The authors found that the incidence is 8 ATR per 100.000 people/year. This is stated both in the results and conclusion sections in the abstract and the manuscript. At the same time the authors discuss the possibility of this number to be an underestimation of the true incidence. I would suggest rephrasing these sentences and just state that you found an incidence of 8 ATR / 100.000 / year, not that it is. | Edited as suggested (Line 55, Line 206) |
| 2) | Minor concern: Line 10-11: The authors claim that the incidence rates range “from 2.5-32.2”.... Suggest to instead use “range from NN to NN” or “the incidence range is NN-NN”. In addition, the authors have not considered the incidence data from Sweden published by Huttunen et al in 2014. They found an incidence of almost 47 ATR / 100000 / year in 2012. Please include this reference and adjust the incidence range accordingly. (Huttunen TT, Kannus P, Rolf C, Felländer-Tsai L, Mattila VM. Acute achilles tendon ruptures: incidence of injury and surgery in Sweden between 2001 and 2012. Am J Sports Med. 2014 Oct;42(10):2419-23. doi: 10.1177/0363546514540599. Epub 2014 Jul 23. PMID: 25056989.) | Edited as suggested (Line 75)  Edited as suggested (Line 76) |
| 3) | Minor concern: From author guidelines: “References are listed at the end of the manuscript and numbered in the order that they appear in the text.” Please comply with the author guidelines. | Edited all in text references and reference list as suggested. |
| 4) | Major concern: Lines 28-37: I will strongly suggest that the authors revise the section in lines 28-37 to adhere to updated strong evidence of increased risk of re-rupture after non-operative treatment. | Using level 1 evidence (systematic review with Meta-analysis https://www.frontiersin.org/articles/10.3389/fsurg.2021.607743/full) we can clearly see that conservative treatment – older style practices akin to that used by Myhrvold et al, is a higher re-rupture rate. However, when we compare accelerated functional rehabilitation methods, we see a similar result for re-ruptures. The regime in Leicester (LAMP) is an accelerated functional rehabilitation method and therefore our discussion is correct. We do accept that this may have been misleading and have modified the manuscript throughout to make this point explicitly throughout our manuscript. |
|  |  |  |
